# A systematic review of medication costs associated with preterm infants

**DOI:** 10.1186/s12962-026-00802-4

**Published:** 2026-07-23

**Authors:** Yunhao Xu, Kamran Khan, Nia Roberts, Sungwook Kim

**Affiliations:** 1 Centre for Health Economics, University of York, York, UK; 2 Warwick Medical School, University of Warwick, Coventry, UK; 3 Bodleian Health Care Libraries, University of Oxford, Oxford, UK; 4 Nuffield Department of Primary Care Health Sciences, University of Oxford, Oxford, UK

**Keywords:** Preterm birth, Economic cost, Medication, Infant, Child

## Abstract

**Background:**

Preterm birth significantly affects neonatal and child health through various severe complications, contributing to high neonatal and under-5 mortality rates globally. While the overall medical costs associated with preterm birth have been explored, comprehensive studies specifically focused on the medication costs related to health conditions arising from preterm birth remain limited.

**Methods:**

Systematic searches were conducted across MEDLINE, Embase, Web of Science, International HTA database and Pediatric Economic Database Evaluation Project using broad search terms for literature published before May 31, 2024. Included studies, published in English, reported the medication costs of preterm birth individuals from birth to age 17 using primary evidence, which were then categorised according to corresponding life stages. Collected medication costs were converted to U.S dollars for the financial year in which the costs were reported based on purchasing power parities and inflated to 2025 U.S. dollar values.

**Results:**

The literature search yielded 5,269 articles. Of these, 42 articles satisfied criteria and were incorporated into this systematic review. Among these articles, 38 papers reported medication costs in infancy (under age 1), 3 papers reported costs in childhood (age 1 to age 12) and 1 paper reported costs across infancy and childhood. The review found that medication costs for common complications varied widely, with higher costs associated with severe conditions and specific treatments. The highest medication cost in this study was observed in patients with chronic lung disease treated with nitric oxide for approximately 24 days, totalling $20,705 per patient (2025 USD). Additionally, costs during childhood showed variability influenced by gestational age at birth and respiratory disease history. In infancy, medication costs typically accounted for less than 15% of total healthcare expenditure in studies reporting both components; childhood evidence was limited and suggested a smaller share of total costs.

**Conclusions:**

This review documented wide variation in medication costs among preterm infants, with the highest costs linked to selected conditions and treatments. Childhood evidence was limited to three heterogeneous studies, and no adolescent data were identified. Future economic evaluations should derive medication-cost parameters from reliable primary sources.

**PROSPERO registration number:**

CRD42024599548.

**Supplementary Information:**

The online version contains supplementary material available at https://doi.org/10.1186/s12962-026-00802-4.

## Background

Preterm birth is defined by the World Health Organization (WHO) as delivery before 37 completed weeks or less than 259 days of gestation [1]. Preterm infants generally stay longer in neonatal intensive care units (NICUs) compared to full-term infants [2]. Throughout their early development in the NICU and beyond, preterm infants often face various health challenges that necessitate the use of a diverse array of medications. These pharmaceutical interventions are not only crucial during their initial hospital stay but may also be required to address ongoing complications that can arise throughout their lives. The spectrum of medications needed can be extensive, reflecting the complex and sometimes long-term health issues associated with premature birth. These medications can be costly, especially when considering the extended duration of NICU care. Respiratory distress syndrome (RDS), a common complication of preterm birth affecting the neonatal respiratory system, often requires surfactant therapy to alleviate the condition [3, 4]. A study from Mexico reported that the mean costs of surfactant for extreme preterm and very preterm neonates were $3,325 and $1,737, respectively (2011 USD) [5]. Another common and life-threatening complication, necrotizing enterocolitis (NEC), requires medication interventions, including withholding enteral feeds with parenteral nutrition support, often extending NICU stays [6]. In one study [7], NEC requiring medication intervention was associated with an additional cost of $13,136 (2009 USD). These examples underscore the substantial medication expenses associated with the care of preterm infants during their NICU stays.

In addition to the high medical costs incurred during the NICU period, preterm birth individuals are also at increased risk of developing long-term health issues. These conditions often require ongoing medical interventions, adding further financial burdens over time. Cerebral palsy (CP) is a typical long-term health issue in preterm babies affecting muscle tone, mobility, and coordination. Various medications including baclofen; diazepam; botulinum toxin; and antispasmodics are frequently used in clinical environments to treat and control symptoms of CP [8]. Although specific data on the medication costs associated with CP are lacking, a study reported the overall lifetime cost of CP per patient in South Korea, underscoring the high healthcare utilisation associated with this condition. The estimated lifetime costs for CP are $26,383 in South Korea, which is lower than in Denmark (approximately $74,000) and the United States (approximately $90,000) [9]. Apart from CP, preterm birth has been consistently associated with an increased risk of chronic conditions such as hypertension, diabetes, lipid disorders, and long-term obstructive lung disease [10]. Managing these conditions often necessitates ongoing monitoring and lifelong medication, further contributing to the long-term healthcare burden for individuals born preterm.

While it is well-established that preterm infants require extensive medical care, including the use of numerous medications, detailed analyses of the medication costs, especially in relation to different life stages and health conditions, are limited. Existing studies predominantly focus on overall medical costs and their association with various preterm birth categories or birth weights, with less emphasis on how medication costs contribute to the total economic burden.

Although the overall financial burden and cost-effectiveness of preterm care are determined by total healthcare expenditures, medication expenses represent a specific and critical component within these broader costs. Currently, a lack of targeted focus on pharmaceutical expenditures leaves a gap in understanding the specific drivers of treatment costs for healthcare systems and families. Without disaggregated data detailing these specific expenses, efforts to understand regional variations and build highly detailed economic models are limited. Therefore, the aim of this study is to systematically review existing global evidence specifically related to the medication costs of preterm individuals, and to conduct a descriptive synthesis to identify patterns within these cost data.

## Methods

### Systematic review

This systematic review followed the Preferred Reporting Items for Systematic Reviews and Meta-Analysis (PRISMA) guideline [11].

### Search strategy

The search strategy was built and piloted with guidance and search expertise provided by Bodleian Health Care Libraries, University of Oxford. Each search term was individually searched to maximise the search results. The final search strategy consisted of controlled-vocabulary terms and free-text search terms associated with preterm birth, medication, infant, children and adolescents and their intersection with costs.

Terms were searched in titles, abstracts and keywords. The search encompassed literature published before 31 May 2024. The final search strategy was applied to several electronic databases: MEDLINE, Embase, Web of Science (Science Citation Index Expanded, Social Sciences Citation Index), International HTA database (https://database.inahta.org) and Pediatric Economic Database Evaluation (PEDE, http://pede.ccb.sickkids.ca/pede/search.jsp) Project.

Since each database has its unique searching rules, search strategies were adjusted accordingly. Beyond adjustments in syntax, the selection of search terms was slightly refined to ensure more accurate and comprehensive retrieval. For MEDLINE, Medical Subject Headings (MeSH), a controlled and hierarchically-organised vocabulary [12], were used to identify corresponding medical concepts and mitigate omissions that might arise from relying solely on free-text and wildcard searches. For Embase and Web of Science, which do not support MeSH terms, the MeSH terms used in MEDLINE were converted to free text and incorporated into the search strategy. In the International HTA database, as it focuses on health technology assessments, terms related to economic costs and life stages were removed to maximise search results. For PEDE, a database dedicated to pediatric economic costs, terms related to costs and life stages were also excluded. Given its limited search functionality and scope, “preterm” and “prematurity” were used as individual search terms. The detailed search strategy is attached in Appendix 1.

All literature identified by the search was imported in Rayyan, which is a web-based application for conducting systematic review, where duplicates were also removed. A two-stage screening process was conducted. At the first stage, titles, abstracts and keywords were evaluated by two independent reviewers (YX and KK). If an article received approval from both reviewers, it proceeded to the second stage. Otherwise, it was referred to a third reviewer for final assessment (SK). At the second stage, full-text articles were reviewed and assessed whether they met the inclusion criteria by YX and KK.

### Eligibility criteria

The main inclusion criteria framing the search strategy were literature reporting medication costs related to preterm born individuals during infancy, childhood and adolescence published in English language and before 31 May 2024. In this study, medication is defined according to the European Medicines Agency as “a substance or combination of substances that is intended to treat, prevent, or diagnose a disease, or to restore, correct, or modify physiological functions by exerting a pharmacological, immunological, or metabolic action.” [13] This definition encompasses not only pharmacologic agents but also prophylactic biologics, skin protectants and medical nutrition. The scope of medication costs is limited specifically to drug acquisition expenses. Medication administration and bundled treatment-pathway costs are not included. The life stages in this study are clearly defined: infants are categorised as individuals under one year of age [14], children as those between one and twelve years of age [15], and adolescents as those aged 13–17 [16]. Studies reporting medication costs for preterm individuals aged over 17 were not included. A comprehensive range of study designs is considered in this study: prospective cohort, retrospective cohort, and cross-sectional studies; randomised controlled trials (RCTs); quasi-randomised controlled trials; cost analysis studies; cost-effectiveness analysis studies; cost-benefit analysis studies; cost-utility analysis studies and budget impact studies.

Studies that reported medication costs based on modelling outcomes (e.g., decision tree, Markov models) were excluded. Commentaries, editorials, study protocols, abstracts, guidelines and debates were excluded. Additionally, grey literature was not considered. Studies that did not specify monetary amounts in currency units or those where costs could not be disaggregated at the patient level were also excluded.

### Data extraction

From each article that met the inclusion criteria, we extracted the following information about its characteristics and entered it into an Excel database: (1) Literature details, including title and authors; (2) Study start and end date; (3) Location of study; (4) Study design; (5) Perspective (the viewpoint from which costs are analysed and assessed); (6) Sample size; (7) Life Stage; (8) Gestational age(s) at birth; (9) Health condition; (10) Medication; 11) Birth weight(s) at birth; 12) Gestational age(s) at birth; 13) Aim of medication; 14) Duration of medication; 15) Cost amount in report; 16) Currency in report; 17) Financial year for which the cost is reported.

For studies that do not report a financial year, it was assumed to be the start year of the study period. If the study period is also not reported, the financial year was assumed to be one year prior to publication. For studies that did not report a specific health condition, preterm birth was recorded as a health condition. Otherwise, reported health conditions were recorded.

Given the diversity of study locations and the various currencies reported, the total medication costs were converted to U.S. dollars for the financial year in which the costs were reported. This conversion utilised the purchasing power parities (PPP) tool published by the OECD [17]. Subsequently, these amounts were updated to reflect 2025 U.S. dollar values using the Consumer Price Index data on medical care provided by the U.S. Bureau of Labor Statistics [18]. The daily medication costs were also calculated when data was available. Medication costs in the main text are rounded to the nearest dollar and study location reported in parentheses. All health conditions recorded in the database were allocated with corresponding International Classification of Diseases 11th Version (ICD-11) codes [19].

### Quality appraisal

Two independent reviewers (YX and KK) assessed the certainty of the evidence for primary outcomes using the GRADE framework [20]. Evidence was graded as high, moderate, low, or very low based on five downgrading criteria (risk of bias, inconsistency, indirectness, imprecision, and publication bias) and three upgrading factors for observational studies (large magnitude, dose-response and all plausible confounding would reduce the demonstrated effect or increase the effect if no effect was observed). By default, randomised clinical trials are assigned a “High” grade (4 out of 4) at the initial stage and observational studies are assigned a “Low” grade (2 out of 4). Disagreements were resolved through consensus or consultation with a third reviewer (SK).

## Results

**Fig. 1:**
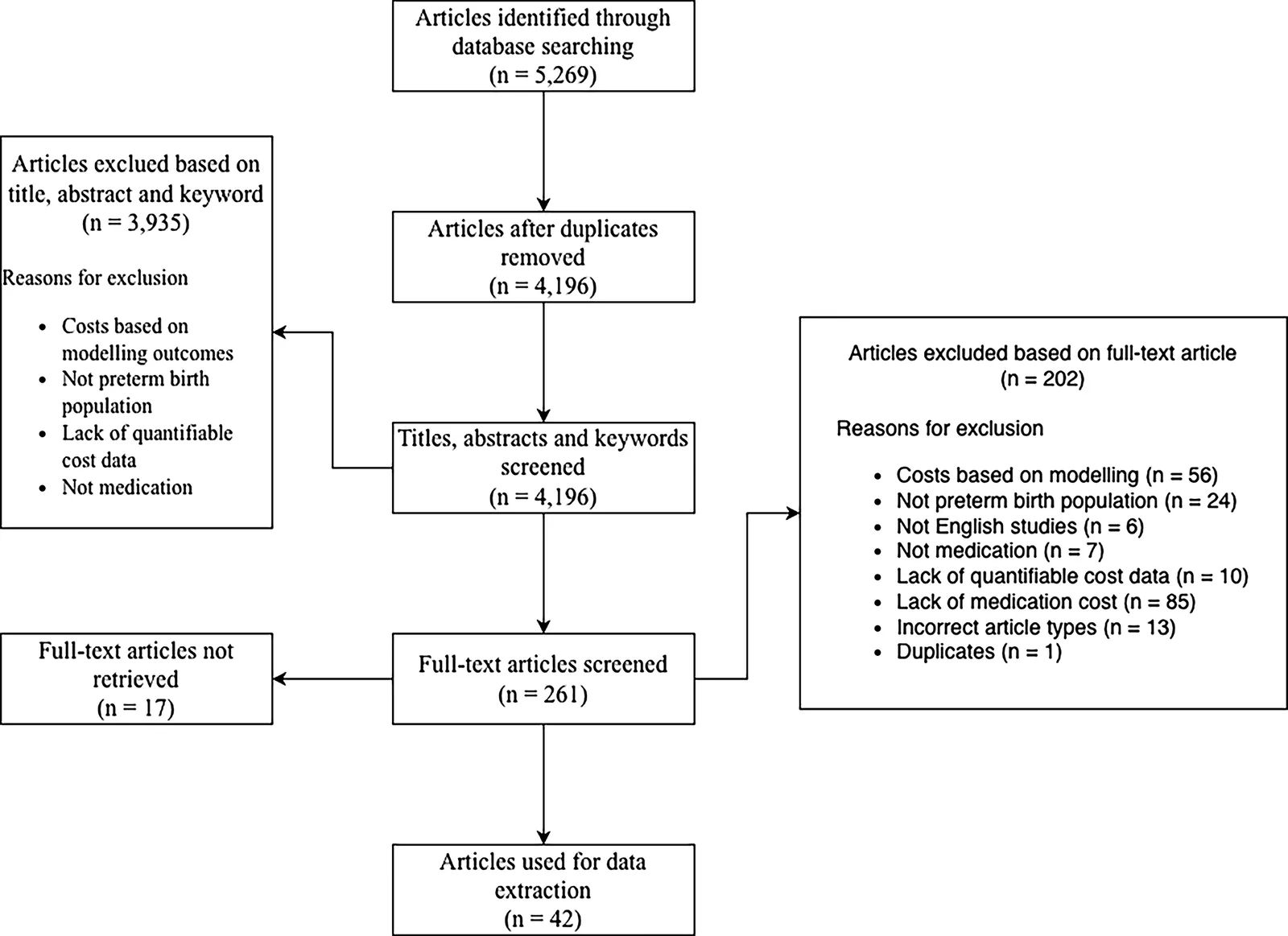
PRISMA flow diagram

A combined literature search yielded a total of 5,269 articles (Fig. 1). 3,023 articles remained after elimination of duplications. Of these, only 234 articles met the inclusion criteria after screening for titles, abstracts and keywords. During the full-text screening process, 15 articles were removed due to lack of access and a further 181 articles were excluded based on the inclusion and exclusion criteria. Ultimately, 42 articles satisfied the inclusion criteria and were incorporated into this systematic review.

As shown in Table 1, cost data cover two life stages: infancy (before age 1) and childhood (age 1–12). Although the search strategy included adolescence (age 13–17), no study reported medication costs during that period. For infancy, this study includes 38 papers [21–58], whereas for childhood, it incorporates 3 papers [59–61]. One paper [62] is included in both the infancy and childhood categories as it provides medication cost data from birth to age 2. Detailed medication costs, such as the type of medication, reported amount, currency, financial year, and the corresponding amount converted to 2025 USD, are succinctly presented in Table 1. More detailed information on study characteristics and medication costs is presented in Appendix 2.

**Table 1:** Medication costs of preterm individuals

ID	Author	Perspective	Life stage	Condition	Medication Name	Aim of Medication	Duration of Treatment	Cost in report	Cost in report per day	Currency	2025 USD
1	Dani et al. (2014)	Hospital	Infant ^1^	Respiratory distress syndrome	Surfactant	Treat	2 doses	Early treatment group: 438.58 Late treatment group: 311.74	Not reported	EUR	Early treatment group: $811.68 Late treatment group: $576.94
2	Kreissl et al. (2016)	Not reported	Infant	Preterm birth	PN solution Numeta	Treat	Not reported	PN solution group mean: 42 Numeta group mean: 62	PN solution group mean: 42 Numeta group mean: 62	USD	PN solution group mean: $71.90 Numeta group mean: $106.14
3	Comas-García et al. (2020)	Hospital	Infant	Respiratory syncytial virus infection	Antibiotic therapy, name of drugs not listed	Treat	Not reported	Control group median: 757.5 RSV infection group median: 1,086.1	Not reported	USD	Control group median: $881.65 RSV infection group median: $1,264.11
4	Hastings et al. (2004)	Not reported	Infant	Respiratory distress syndrome	Beractant Calfactant	Prevent Treatment	Not reported	Prophylaxis group - Beractant mean: 379.39 Prophylaxis group - Calfactant mean: 512.72 Rescue group - Beractant mean: 689.56 Rescue group - Calfactant mean: 772.40	Not reported	USD	Prophylaxis group - Beractant mean: $806.76 Prophylaxis group - Calfactant mean: $1,090.29 Rescue group - Beractant mean: $1,466.33 Rescue group - Calfactant mean: $1,642.49
5	McCallum and Turbeville (1994)	Not reported	Infant	Intraventricular haemorrhage	Not reported	Treat	Initial hospitalisation	11,108.00	Not reported	USD	$43,159.90
6	Hernández-Gago et al. (2017)	Health system	Infant	Respiratory syncytial virus infection	Palivizumab	Prevent	Not reported	08/09: 2,745.28 09/10: 2,579.68 10/11: 2,165.03 11/12: 2,130.03 12/13: 2,402.62 13:14: 2,894.62	Not reported	EUR	08/09: $6,026.40 09/10: $5,543.46 10/11: $4,447.18 11/12: $4,322.88 12/13: $4,833.38 13:14: $5,853.22
7	Phibbs et al. (1993)	Hospital	Infant	Respiratory distress syndrome	Exosurf	Treat	Single dose	900	Not reported	USD	$3,206.95
8	Desgualdo et al. (2011)	Hospital	Infant	Preterm birth	Not reported	Treat	Initial hospitalisation	< 1,000 g group: 388 1,000–1,499 g: 332 1,500–1,999 g: 69 2,000–2,499 g: 36 > 2,500 g: 27 Mean medication cost: 121	Not reported	USD	Average medication cost: $226.35
9	Geraerds et al. (2021)	Hospital	Infant	Early onset sepsis	Antibiotic treatment	Treat	74 h	PCT-guided decision-making group mean: 22 Standard care group mean: 26	Not reported	EUR	PCT-guided decision making group mean: $35.27 Standard care group mean: $41.68
10	Mugford et al. (1991)	Not reported	Infant	Respiratory distress syndrome	Not reported	Treat	Initial hospitalisation	Respiratory distress syndrome group: 199.65 Without respiratory distress syndrome group: 29.20	Not reported	GBP	Respiratory distress syndrome group: $1,279.28 Without respiratory distress syndrome group: $187.10
11	King et al. (2021)	Hospital	Infant	Preterm birth	Not reported	Treat	Initial hospitalisation	Median estimated cost: 4,109	Not reported	USD	$4,917.69
12	Sandhu et al. (1986)	Not reported	Infant	Very low birth weight	Not reported	Treat	Initial hospitalisation	156.25	Not reported	GBP	$1,604.04
13	Tubman et al. (1990)	Not reported	Infant	Respiratory distress syndrome	Surfactant	Treat	Initial hospitalisation	Treatment group: 1156	Not reported	GBP	Treatment group: $10,918.14
14	Johnson et al. (2015)	Not reported	Infant	Necrotizing enterocolitis, very low birth weight	Not reported	Treat	Initial hospitalisation	With NEC group mean: 11,900 Without NEC group mean: 5,080	Not reported	USD	With NEC group mean: $16,637.3 Without NEC group mean: $7,102.31
15	Lewis et al. (2023)	Hospital	Infant	Low birth weight	Prescription medication	Treat	Not reported	Treatment group mean: 4,511 Control group mean: 2,223	Not reported	USD	Treatment group mean: $5,250.34 Control group mean: $2,587.35
16	Soll et al. (1993)	Hospital	Infant	Respiratory distress syndrome	Beractant Sham-air	Treat	initial hospitalization	Beractant group mean: 1,375 Sham-air group mean: 1,853	Beractant group mean: 54 Sham-air group mean: 80	USD	Beractant group mean: $4,506.44 ($180.26 daily) Sham-air group mean: $6,073.05 ($264.05 daily)
17	Vogel et al. (2002)	Societal	Infant	Respiratory syncytial virus infection	Palivizumab	Prevent	Not reported	6,563.00	Not reported	NZD	$16,394.42
18	Petrou et al. (2021)	NHS and PSS	Infant	Low birth weight	Not reported	Treat	Not reported	Intervention group mean: 79 Control group mean: 78	Not reported	GBP	Intervention group mean : $143.53 Control group mean: $141.72
19	LeFevre et al. (2010)	Provider	infant	Preterm birth	Sunflower seed oil, Aquaphor, Injections of antibiotics, intravenous saline, syrups and other medications	Prevent	Not reported	Drug: SSO group mean: 8.02 Aquaphor group mean: 7.40 Control group mean: 7.90 Emollient: SSO group mean: 1.55 Aquaphor group mean: 29.00 Control group mean: None	Not reported	USD	Total cost of emollient and drug SSO group mean: $16.51 Aquaphor group mean: $62.80 Control group mean: $13.63
20	Petrou et al. (2009)	Public sector	Child	Extreme preterm birth	Not reported	Treat	12 month at age 11	23.7	Not reported	GBP	$58.71
21	Ogata et al. (2016)	Not reported	Infant	Preterm birth	Not reported	Treat	Not reported	With ACS group mean: 225 Without ACS group mean: 321	Not reported	USD	With ACS group mean: $307.02 Without ACS group mean: $438.01
22	Ganapathy et al. (2012)	Not reported	Infant	Necrotizing enterocolitis	mother’s milk fortified with bovine milk based human milk fortifier, mother’s milk fortified with a donor human milk based human milk fortifier	Prevent	During NICU	BOV arm mean: 195 HUM arm mean: 10,321	Not reported	USD	BOV arm mean: $282.62 HUM arm mean: $14,958.43
23	Wegner et al. (2004)	Payer	Infant	Respiratory syncytial virus infection	Palivizumab	Prevent	Not reported	Average seasonal cost of palivizumab infection: 4996.46	Not reported	USD	$9,755.83
24	Achten et al. (2020)	Hospital	Infant	Early onset sepsis	Antibiotic	Treat	Initial hospitalisation	Before implementation: 1.45 After implementation: 0.64	Not reported	EUR	Before implementation: $2.26 After implementation: $1.00
25	Dukhovny et al. (2011)	Third-party	Infant	Apnea of prematurity	Caffeine	Treat	Based on the discretion of the provider	Caffeine cost mean: 76	Not reported	CAD	$98.10
26	Zupancic et al. (2009)	Third-party	Infant	Chronic lung disease	Nitric oxide	Treat	minimum of 24 days	Nitric oxide cost: 12,000	Not reported	USD	$20,705.60
27	McBride et al. (2009)	Third-party	Infant	Chronic lung disease	Recombinant human superoxide dismutase (rhSOD)	Prevent	28 days of life	SOD group mean: 3,550 Control group mean: 3,350	Not reported	USD	SOD group mean: $6,933.88 Control group mean: $6,543.23
28	Ohls et al. (1995)	Not reported	Infant	Very low birth weight	Recombinant human erythropoietin and erythrocyte (Epo)	Treat	14 days	Epo cost: 129	9.21*	USD	$354.66 ($25.33 daily)
29	Dallas et al. (1998)	Not reported	Infant	Very low birth weight	Glutamine supplementation	Treat	27 days	Glutamine supplementation cost: 810*	30.00	USD	$2,471.77 ($91.55 daily)
30	Ngabonzima et al. (2022)	Healthcare payer	Infant	Preterm birth	Not reported	Treat	Initial hospitalisation	7,766.30	Not reported	USD	$8,576.91
31	Blake et al. (2017)	Not reported	Infant	Respiratory syncytial virus infection	Palivizumab	Prevent	3 doses	4,500	Not reported	USD	$5,629.93
32	Greenough et al. (2004)	Not reported	Child ^2^	Preterm birth with RSV infection history	Not reported	Treat	years 2–4	RSV proven median: 40 Non-RSV median: 27	Not reported	GBP	RSV proven median: $117.77 Non-RSV median: $79.49
33	Levin et al. (2021)	Third-party	Infant, Child	Preterm birth	Not reported	Treat	year 0–2	1,888	Not reported	USD	$2,110.78
34	Yang et al. (2023)	NHS	Infant	Very preterm birth	Nitric oxide, surfactant, palivizumab	Treat	Initial hospitalisation	Nitric oxide cost: 48.2* Surfactant cost: 630.18* Palivizumab cost: 71.84*	Not reported	GBP	Nitric oxide cost: $83.88 Surfactant cost: $1,096.68 Palivizumab cost: $125.02
35	Field et al. (2005)	Not reported	Infant	Severe respiratory failure	Nitric oxide	Treat	Initial hospitalisation	Nitric oxide cost mean: 1,777	Not reported	GBP	$5,231.93
36	Petrou et al. (2006)	Societal	Infant	Extreme preterm birth	Not reported	Treat	One year	10.00	Not reported	GBP	$28.06
37	Uko et al. (2006)	Not reported	Infant	Late-onset invasive fungal infection	Fluconazole	Prevent	Initial hospitalisation	124.85*	Not reported	USD	$243.78
38	Cömert et al. (2012)	Not reported	Infant	Preterm birth	Not reported	Treat	Initial hospitalisation	180.95*	Not reported	USD	$312.22
39	Chandra et al. (2023)	Not reported	Infant	Neonatal sepsis	Antibiotic therapy	Treat	Initial hospitalization	Antibiotics: 14 Total drug cost: 303	Not reported	USD	Antibiotics: $14.86 Total drug cost: $321.60
40	Cherian et al. (2017)	hospital	Infant	Respiratory distress syndrome	Surfactant	Treat	Initial hospitalisation	Surfactant costs: GA 28–31 weeks: 203 GA 32–36 weeks: 189	Not reported	USD	Surfactant costs: GA 28–31 weeks: $247.75 GA 32–36 weeks: $230.66
41	Roggeri et al. (2016)	Italian NHS	Infant	Bronchiolitis	Not reported	Treat	Initial hospitalisation	First year: 58 Second year: 153 Third year: 50 Fourth year: 65	Not reported	EUR	First year: $101.95 Second year: $268.95 Third year: $87.89 Fourth year: $114.26
42	Geitona et al.	Greece NHS	Infant	extreme preterm birth, very preterm birth, preterm birth	Not reported	Treat	Initial hospitalisation	GA < 32 weeks: 45,299 GA 32–37 weeks: 11,139	Not reported	EUR	GA < 32 weeks: $121,829.11 GA 32–37 weeks: $30,004.08

* Figures calculated from the data provided. Detailed information is included in Appendix 2

^1^An infant is defined as an individual under age 1

^2^A child is defined as an individual between the ages of one and twelve

Within the 42 identified studies, the certainty of evidence was appraised as high for 11 publications, while moderate, low, and very low grades were assigned to 14, 15, and 2 studies, respectively. Notably, three observational studies were elevated to a high-quality grade; these investigations employed quasi-experimental methodologies, such as propensity score matching, to mitigate potential confounding variables and demonstrated substantial effect sizes. Conversely, eight studies utilising RCT designs were downgraded due to indirectness stemming from the use of outdated data (prior to 2000) or imprecision related to small sample sizes of fewer than 20 participants. Detailed outcomes of quality appraisal are presented in Appendix 3.

### Methodological characteristics

Geographically, 16 studies were conducted in the United States [24, 25, 31, 34–36, 41, 42, 44–47, 49, 53, 62, 63], 3 in Latin America [23, 28, 40], 11 in Europe [21, 22, 26, 30, 32, 33, 43, 50, 57, 58, 61], 4 in Asia [39, 54–56], 1 in Oceania [37], 1 in Africa [48] and 6 studies [29, 38, 51, 52, 59, 60] were based on multiple countries. Among 42 included papers, only 16 papers were published within 10 years.

From a study design perspective, 14 studies are randomised controlled trials [21, 30, 33, 36–39, 41, 44–47, 51, 60] and the remaining studies can be broadly categorised as observational studies.

This systematic review includes several different cost perspectives. The majority of studies did not report their cost perspective. Among those that did, 2 studies used a societal perspective [37, 52], 6 studies used a third-party payer perspective [42, 44, 45, 48, 60, 62], and the remaining studies used either a healthcare system perspective or a hospital perspective.

### Medication costs during infancy

The majority of studies included in this review reported the range or mean of gestational age from included infants. Only four papers just mentioned their medication costs were based on the preterm infant group instead of providing gestational age-related statistics. The most common health conditions addressed by the included studies were very low birth weight (VLBW), respiratory syncytial virus (RSV) infection, RDS, intraventricular haemorrhage (IVH), and NEC, all of which are typical complications associated with preterm birth [64]. Regarding the financial years of the included studies, 8 studies were conducted or utilised data collected before 2000, 14 studies between 2000 and 2010, and 16 studies after 2010.

Based on the data aggregated in Table 1, RSV prophylaxis is frequently mentioned using palivizumab. Four studies [37, 42, 49, 65] included in this review reported its mean cost ranging from $4,322 (Spain) to $16,394 (New Zealand) per person for different protocols (2025 USD). The dosage of palivizumab is determined by the infant’s weight at the time of prophylaxis, which can vary significantly depending on gestational age at birth and prevention protocol. This variation in weight directly influences the medication costs, leading to potentially significant differences across studies. RDS as a common respiratory complication also associated with high medication costs and surfactant is used for prevention or treatment. Seven studies [21, 24, 27, 33, 36, 50, 56] reported its mean cost ranging from $230 (India) to $10,918 (UK) per person for unstated duration (2025 USD). Among these, five studies reported the mean costs for initial hospitalisation, without specifying the duration of stay, while two studies reported costs based on the dosages used, also without indicating duration. The cost for one dose of Exosurf was $3,206 (US), and the cost for two doses of surfactant ranged from $576 (Denmark) to $811 (Denmark) per person (2025 USD), depending on the weight at the time of drug administration. Apart from RSV infection and RDS, chronic lung disease and severe respiratory failure were also reported in the included studies. Both conditions utilised nitric oxide as a treatment. For chronic lung disease, the average medication cost was $20,705 (US) per person for 24 days or longer treatment (2025 USD) [44], while for severe respiratory failure, the mean cost was $5,231 (UK) per person during initial hospitalisation, with the length of stay unspecified (2025 USD) [51]. Additionally, Dukhovny et al. [60] reported the mean medication cost of caffeine as $98 (multiple countries) per person. The duration of caffeine was based on the discretion of providers, with caffeine initiated to be administered during the first ten days of life to treat apnea of prematurity in preterm individuals. NEC is a life-threatening complication of preterm birth. Two studies included in this review reported the costs associated with its treatment and prophylaxis. A cost-effectiveness analysis (CEA) [34] conducted in the United States revealed that the medication cost for NEC treatment during initial hospitalisation was $16,637 (US) per person, with the length of stay unspecified (2025 USD). Another prophylactic study [41] compared the medication costs of two interventions: mother’s milk fortified with bovine milk-based human milk fortifier (HMF) and mother’s milk fortified with donor human milk-based HMF. The reported mean costs were $282 (US) and $14,958 (US) per person during their NICU stay, with the length of stay unspecified (2025 USD), respectively. The significant cost disparity is attributed to the extremely high cost of human milk-based HMF, priced at $6.25/mL (2011 USD), with a median volume of 1,650.8 mL required for a preterm infant. Studies focused on birth weight were also included in this review. Two studies reported medication costs for low-birth-weight infants. One [66] compared mean medication costs between infants with and without home visits, finding higher costs for the home visit group at $5,250 (US) compared to $2,587 (US) per person from discharge after initial hospitalisation until age 1 (2025 USD). Another study [38] focused on real-time continuous glucose monitoring for glucose control among low-birth-weight infants, reporting similar mean medication costs for the intervention and control groups, around $140 per person for the first seven days after birth (2025 USD).

Only a few studies in this review provided daily medication costs for specific treatments. One study [22] reported the costs of two nutrient solutions for preterm infants: $72 (Austria) per day for parenteral nutrition and $106 (Austria) per day for Numeta (2025 USD). Another study [36] compared daily costs for Beractant and sham-air in treating RDS, which were $180 (US) and $264 (US), respectively (2025 USD). Additionally, two studies [36, 47] highlighted the costs of recombinant human erythropoietin and erythrocyte supplementation at $25 (US) per day, and glutamine supplementation at $91 (US) per day, both aimed at treating very low birth weight infants (2025 USD).

### Medication costs during childhood

The three studies all involved comparisons based on characteristics of the study groups. Greenough et al. [61] compared the medication costs between very preterm birth (VPT) children and VPT children with a history of RSV during ages 2–4. The median three-year medication cost per VPT children was $37 (UK) accounting for 1.9% of total healthcare cost during this period, while VPT children with a history of RSV infection had a slightly higher average cost at $56 (UK) but accounted for a lower percentage (1.5%) of total healthcare cost as these children have a much higher inpatient cost (2025 USD). Petrou et al. [59] evaluated the mean cost of 12-month medication for each extreme preterm birth survivor at age 12, estimating it to be $28 (UK and Ireland) (2025 USD), which accounted for less than 1% of total cost. In addition, Levin et al. [62] documented the medication costs for preterm individuals during their first two years of life, spanning both infancy and childhood. This cost, which includes both out-of-pocket expenses and amounts paid by insurers, was reported as $2,110 (US) per person for the first two years of life (2025 USD). In summary, medication costs for preterm individuals during childhood are influenced by gestational age at birth and respiratory disease history.

## Discussion

This study systematically reviewed the medication costs of preterm birth individuals during their infancy and childhood and conducted an analysis of 42 studies, revealing significant variability across different conditions and life stages. The analysis found that medication costs for common complications such as RDS, VLBW, and NEC varied widely, with higher costs associated with severe conditions and specific treatments like palivizumab and surfactant therapy. Additionally, medication costs during childhood showed variability influenced by factors such as gestational age and respiratory history.

Even though the studies included in this review reported medication costs for some preterm birth complications, these represent only a small portion of the health conditions associated with preterm birth, as defined by the Global Burden of Disease [67]. Several factors could explain this limited scope. First, some health conditions are uncommon in clinical practice [68–70], such as Wilson-Mikity syndrome, a chronic lung disease affecting premature infants. The limited study populations for such rare conditions likely contribute to the scarcity of medication cost data. Additionally, conditions with extremely high mortality rates may be less appealing for researchers to explore from a cost perspective, as these cases often focus more on survival outcomes than on detailed economic analyses. Moreover, in severe health issues such as congenital abnormalities and retinopathy of prematurity, medication is often not the primary treatment. This is due to the lack of corresponding medications or the possibility that medications may take a long time to be effective, potentially exacerbating the conditions [71–74]. These conditions typically require surgical interventions, and as a result, research studies tend to focus on surgical or overall medical costs rather than breaking down the expenses related specifically to medication. The lack of detailed cost data for these conditions underlines the need for more focused research in this area, especially on the cost of rare but severe preterm birth-related complications.

Some studies in this review also reported overall medical costs, with medication expenses accounting for only a small fraction of the total. In most of these studies, medication costs during infancy typically comprised less than 15% of the total healthcare expenditure [30, 31, 34, 36]. The largest proportion of costs was attributed to care-related expenses, such as room charges and staffing, which frequently accounted for more than 75% of the overall costs [34]. Equipment costs, particularly for infants suffering from respiratory conditions who require ventilators, also contributed significantly to total costs, often exceeding medication expenses [30, 36]. This pattern suggests that, for most preterm infants, medication costs alone are unlikely to be the primary driver of NICU budget pressure, and policies aimed solely at drug price reduction may have limited impact on overall NICU affordability unless they also affect length of stay, complication rates, or non-drug resource use.

Regarding RSV prophylaxis policy, the wide geographic variation in palivizumab costs underscores that policy effectiveness depends not only on clinical eligibility but also on dosing schedule, local price, and implementation strategy [37, 42, 49, 65]. Jurisdictions considering restricted versus expanded prophylaxis should incorporate country-specific unit costs, number of doses, and expected eligible population size into budget-impact analysis. The present review provides real-world cost inputs for such analyses but cannot, by itself, determine cost-effectiveness; that requires linking these costs to effectiveness estimates and local epidemiology.

This review has implications for parameter sourcing in health technology assessment (HTA) modelling. Many included studies reported condition-specific or product-specific medication costs derived from primary clinical data, making them suitable inputs for budget-impact models, CEAs, and model-based economic evaluations focused on NICU pathways. By contrast, model-based studies using hypothetical dosing, assumed weights, or manufacturer list prices were excluded because they may over- or under-estimate real-world medication costs. For HTA modellers, our findings suggest three practical rules: (1) prefer primary-cost studies over assumed drug costs where available; (2) report costs by gestational age, birth weight, treatment duration, and indication because pooled averages mask policy-relevant heterogeneity; and (3) explicitly separate drug costs from total NICU costs to avoid double-counting or misattributing the main drivers of expenditure. Important gaps remain for HTA, including adolescent medication costs, rare complications, outpatient and lifelong pharmacotherapy, and standardised reporting of daily drug costs and drug share of total expenditure.

The principal strength of this study lies in its comprehensive cataloguing of medication costs for preterm birth individuals during infancy and childhood. This approach offers a detailed list of medications used on preterm individuals and their corresponding health conditions. Additionally, the medication cost data presented in this review were derived from primary evidence, providing a more accurate reflection of actual medication costs. During the screening process, several CEAs based on decision-analytic models were encountered, which often utilised hypothetical parameters for estimating medication costs. These parameters were typically derived from drug instructions or expert opinions rather than clinical trial data. However, in many CEAs, the infant’s weight or dosage is often assumed based on hypothetical scenarios, which may not accurately reflect the diversity of authentic situations. Although well-designed CEAs incorporate sensitivity analyses to address uncertainty and ensure the robustness of their findings, the hypothetical nature of these parameters makes them unsuitable for a systematic review focused on actual medication costs. As a result, these studies were excluded from this review to maintain the integrity and relevance of the cost data. Furthermore, the classification of data into infancy and childhood allows readers to easily access the information most relevant to their interests. This review also discloses significant variability across different conditions and life stages, providing a nuanced understanding of the medical needs and associated costs throughout the early life of preterm individuals.

This study is subject to several limitations. First, although medication costs have been converted to 2025 USD using PPP and medical-price inflation adjustments, the heterogeneity of study contexts makes it challenging to compare medication costs across most of the included studies or to further analyse the cost variations observed for the same medications or conditions. Second, this study is solely based on narrative synthesis. The heterogeneity in study designs and backgrounds further complicated the possibility of performing a meta-analysis. Despite one method that allows for conducting meta-analysis using the outcomes of quality assessments from each study as weights [75], rather than standard deviations, it still requires developing an appropriate quality assessment tool and addressing the heterogeneity among studies, which is time-consuming. Third, although the GRADE framework was employed for quality appraisal, this tool primarily evaluates the certainty of the evidence rather than the rigor of the underlying costing methodologies, such as analytical perspective, the comprehensiveness of cost items, or valuation techniques. Fourth, the search strategy used in this review could be enhanced by replacing generic medication-related search terms with specific chemical compositions and trade names listed in the British National Formulary for Children (BNFC) [76]. This approach would greatly reduce the number of papers meeting the inclusion criteria being omitted and provide a more structured approach to findings. Additionally, the literature search concluded in 2024 and only included published English literature. The exclusion of grey literature, non-English publications, and recent evidence from late 2024 onwards may potentially introduce selection bias, skewing the reported outcomes toward higher-income clinical settings with elevated costs.

## Conclusions

This research conducted a systematic review of the economic impact of medications on preterm birth individuals during their infancy and childhood (birth to age 12). The analysis found that medication costs for common complications such as RDS, VLBW, and NEC varied widely, with higher costs associated with severe conditions and specific treatments like palivizumab and surfactant therapy. Additionally, while medication costs during infancy were substantial, costs during childhood showed variability influenced by factors such as gestational age and respiratory history. To enhance the reliability and comparability of research on the economic costs of medications associated with preterm birth, future research would benefit from utilising evidence from RCTs or registry databases to better inform economic evaluations of medication costs.

## Supplementary Information

Below is the link to the electronic supplementary material.

Supplementary Material 1

Supplementary Material 2

Supplementary Material 3

## Data Availability

All data generated or analysed during this study are included in supplementary material.

## Abbreviations

WHO: World Health Organization
NICU: Neonatal intensive care unit
RDS: Respiratory distress syndrome
NEC: Necrotizing enterocolitis
CP: Cerebral palsy
PRISMA: Preferred reporting items for systematic reviews and meta-analysis
PEDE: Pediatric economic database evaluation
MeSH: Medical subject heading
PPP: Purchasing power parities
ICD−11: International classification of diseases 11^th^ version
VLBW: Very low birth weight
RSV: Respiratory syncytial virus
IVH: Intraventricular haemorrhage
CEA: Cost-effectiveness analysis
HMF: Human milk fortifier
VPT: Very preterm birth
BNFC: British national formulary for children
RCT: Random clinical trial
HTA: Health technology assessment
